# Cutaneous Anthrax Outbreak in the Trakya Region of Turkey

**DOI:** 10.4274/balkanmedj.galenos.2019.2018.12.19

**Published:** 2019-05-10

**Authors:** Figen Kuloğlu, Alper Akın Gözübüyük, Mehmet Kara, Filiz Akata

**Affiliations:** 1 Department of Infectious Diseases, Trakya University School of Medicine, Edirne, Turkey

To the Editor,

A 34-year-old female presented to our hospital with high fever (39.5 °C), a dark brown lesion on the dorsal side of her right hand, erythema on the medial side of her right arm extending from her right hand to axilla, right axillary lymphadenopathy, and pain in her right arm. She had a history of contact dermatitis on her hands for many years before she came to Enez for a vacation ten days ago. She bought ground meat from a butcher shop, and two days after handling the raw meat, a small pruritic papule appeared on the dorsal side of her right hand. The lesion progressed to a dark brown vesicle with a depressed black necrotic center surrounded by edema (Figure 1). Although she had been prescribed amoxicillin-clavulanate for two days by her family physician, the patient continued to have high fever, and an erythema appeared on the medial side of her right arm. Her white blood cell count was 13.640/mm3, with 76% polymorphonuclear leukocytes and 18% lymphocytes. After thorough examination, she was hospitalized with a clinical diagnosis of cutaneous anthrax with lymphangitis. Vesicular fluid was aspirated for Gram stained smear and culture examination, and 20 million units of Penicillin G was started. After 24 h of treatment, fever and lymphangitis resolved. No microorganism could be detected in the Gram stained smear of the vesicular fluid, and there was no growth on culture medium. After four days of parenteral treatment, the patient was discharged with oral ciprofloxacin therapy for next seven days, and a written informed consent was obtained from the patient.

A day after the first case, a 60-year-old female patient was admitted to the hospital with the clinical diagnosis of cutaneous anthrax. When the patient was questioned, we found that her family slaughtered cattle for dinner in their daughter’s wedding 10 days ago. When we found that the other members of the family (two men and two women) had come into contact with the meat of an infected animal and had similar complaints as those of the patient, we immediately reported the incident to the Public Health Department and Provincial Directorate of Ministry of Food, Agriculture and Livestock, Edirne, to investigate the infected meat and the people who were exposed to the meat. Edirne, Enez, Hisarlı village was quarantined by the Directorate of Public Health and Provincial Directorate of Ministry of Food, Agriculture and Livestock of Edirne. The entry and exit of animals to the region was prohibited. The territory, where the diseased animals were slaughtered, and the butcher shop were taken under control, cleaned up, and decontaminated. The registered animals were controlled, and anthrax vaccine was administered. Samples of meat and swabs were taken from the meat in the patient’s house, meat at the butcher shop, and meat mincer and were sent to the Veterinary Control Institute of Pendik. All the remaining meat was destroyed. Microscopic examination of meat revealed gram-positive bacilli, and *Bacillus anthracis* was evident in the culture growth.

Anthrax is a zoonotic disease endemic in Turkey (1,2). During the four years 2010-2013, six patients with anthrax (five patients with cutaneous anthrax and one with severe sepsis) were treated at Trakya University Hospital (unpublished data). There was no reported case of anthrax in 2014; however, in August 2015, seven patients with cutaneous anthrax were admitted to the hospital. Among them, five had slaughtered cattle and chopped meat for the wedding dinner. The first patient mentioned above, admitted to the hospital with cutaneous anthrax, had come to Enez for a vacation and bought ground meat from a butcher shop, unaware that the meat she bought was of infected cattle. She was the sixth victim of the outbreak.

Anthrax is primarily a disease of grazing herbivores, cattle, and sheep in Turkey (1,2). Naturally acquired cases in humans are usually associated with exposure to anthrax-infected animals and animal products. Majority of the human cases (95.8%) are diagnosed with cutaneous anthrax. *B. anthracis*, the causative agent of anthrax, is a large non-motile spore-forming gram-positive bacillus. Penicillin, used as the first-choice treatment for anthrax, is still effective in Turkey (1,2,3,4). Bacteremia secondary to cutaneous anthrax may lead to seeding of the bacilli in the central nervous system or lungs. Hospitalization is warranted for all the patients with cutaneous anthrax, with signs of systemic involvement, bacteremia, anthrax meningitis, gastrointestinal anthrax, or inhalation anthrax. As these clinical forms are life-threatening, early clinical diagnosis and administration of antibacterial drugs are critical for better survival (1,2,3,4). Sepsis and central nervous system infection, with resultant hemorrhagic meningoencephalitis, are always fatal, as experienced in our hospital in the year 2005 (5) and 2013 (unpublished data).

In conclusion, uncontrolled slaughtering of diseased animals may cause serious public health problems. The most important thing about this outbreak was the growth of *B. anthracis* in the cultures of meat samples that were taken from the meat at the butcher shop, the meat mincer, and the meat in the house. Destruction of the meat of the diseased animal prevented a larger outbreak.

## Figures and Tables

**Figure 1:**
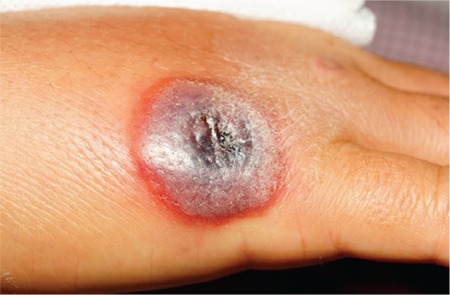
Anthrax lesion in the first patient; a hyperemic, ulcerated lesion with central necrosis surrounded by edema.
